# The p53 Inhibitor MDM2 Facilitates Sonic Hedgehog-Mediated Tumorigenesis and Influences Cerebellar Foliation

**DOI:** 10.1371/journal.pone.0017884

**Published:** 2011-03-18

**Authors:** Reem Malek, Jennifer Matta, Natalie Taylor, Mary Ellen Perry, Susan M. Mendrysa

**Affiliations:** 1 Department of Basic Medical Sciences, School of Veterinary Medicine, Purdue University, West Lafayette, Indiana, United States of America; 2 Laboratory Animal Sciences Program, National Cancer Institute, National Institutes of Health, Frederick, Maryland, United States of America; 3 Laboratory of Protein Dynamics and Signaling, National Cancer Institute, National Institutes of Health, Frederick, Maryland, United States of America; Yale Medical School, United States of America

## Abstract

Disruption of cerebellar granular neuronal precursor (GNP) maturation can result in defects in motor coordination and learning, or in medulloblastoma, the most common childhood brain tumor. The Sonic Hedgehog (Shh) pathway is important for GNP proliferation; however, the factors regulating the extent and timing of GNP proliferation, as well as GNP differentiation and migration are poorly understood. The p53 tumor suppressor has been shown to negatively regulate the activity of the Shh effector, Gli1, in neural stem cells; however, the contribution of p53 to the regulation of Shh signaling in GNPs during cerebellar development has not been determined. Here, we exploited a hypomorphic allele of *Mdm2* (*Mdm2^puro^*), which encodes a critical negative regulator of p53, to alter the level of wild-type MDM2 and p53 *in vivo*. We report that mice with reduced levels of MDM2 and increased levels of p53 have small cerebella with shortened folia, reminiscent of deficient Shh signaling. Indeed, Shh signaling in *Mdm2*-deficient GNPs is attenuated, concomitant with decreased expression of the Shh transducers, Gli1 and Gli2. We also find that Shh stimulation of GNPs promotes MDM2 accumulation and enhances phosphorylation at serine 166, a modification known to increase MDM2-p53 binding. Significantly, loss of MDM2 in *Ptch1^+/−^* mice, a model for Shh-mediated human medulloblastoma, impedes cerebellar tumorigenesis. Together, these results place MDM2 at a major nexus between the p53 and Shh signaling pathways in GNPs, with key roles in cerebellar development, GNP survival, cerebellar foliation, and MB tumorigenesis.

## Introduction

The cerebellum is a highly organized structure that coordinates motor, linguistic and cognitive functions [1]. The internal granular layer (IGL) of the cerebellum contains the majority of the neurons of the brain, and its proper organization requires precise control of both the extent and timing of a number of intricate processes, including the migration, proliferation, and differentiation of granular neuronal precursors (GNPs) [2]. In mice, GNPs are derived from the rhombic lip region of the fourth ventricle prior to embryonic day 14.5 and migrate dorsally over the cerebellar primordium to form the nascent external granular layer (EGL) [3]. The development of the cerebellum continues during the first weeks (mice) or months (humans) after birth, during which time GNPs continue to proliferate prior to their differentiation into mature granule cell (GC) neurons [4], [5]. Improper GNP maturation can result in defective foliation, which is associated with defects in motor coordination [6], [7], or in medulloblastoma, the most common childhood brain tumor [8].

Mitogenic signaling by the Sonic Hedgehog (Shh) pathway is critical for GNP proliferation and expansion of the EGL in the post-natal period of cerebellar development [9], [10], [11]. During this time, Shh is secreted by Purkinje cells and binds to its receptor, Patched (Ptch), which is expressed on GNPs [12]. In the absence of Shh, Ptch inhibits the signaling activity of Smoothened (Smo). Binding of Shh to Ptch alleviates Smo repression, thereby triggering activation of the Gli (Gli1, 2, and 3) family of transcription factors [13], [14]. The proliferative effects of Shh are likely mediated through the transcriptional regulation of Shh target genes that include several transcription factors (e.g. NMyc) [15] and downstream cell cycle regulatory proteins (e.g. CyclinD1) [16]. Shh signaling is down-regulated in GNPs at the later stages of cerebellar development through a mechanism which is not well understood, and this failure of GNPs to respond to Shh correlates with their cell cycle exit and subsequent differentiation into mature GC neurons that migrate inward to form the IGL [4], [5].

Aberrantly constitutive Shh signaling has been implicated in the etiology of several cancers, including medulloblastoma (MB) [17], [18], [19], [20], [21]. One subtype of MB is presumed to arise following the dysregulation of the normal developmental program of a GNP [22]. In agreement with this view, Shh signaling is aberrantly activated in >60% of MB tumors [23]. Ptch1, an inhibitory component of Shh signaling, acts as a tumor suppressor [24] and germ-line mutations in *Ptch1* promote MB formation in both humans and mice [17], [18]. In MB tumors that form in *Ptch1^+/−^* mice, Shh signaling is constitutively active and blocking this signal with a Shh-antagonist promotes tumor regression [25], [26], highlighting the importance of Shh signaling in the initiation and maintenance of this tumor type. Despite the prevalence of Shh dysregulation in MB, mutations in known components of the Shh signaling pathway have been identified in only ∼10–25% of sporadic human MB [27], leaving the genetic basis for most MB tumors unexplained. This gap in knowledge may be filled through the identification of other genes and pathways that collaborate with Shh signaling in GNP maturation.

The p53 pathway is a leading candidate collaborator with Shh in both cerebellar development and MB. A role for p53 inactivation in MB pathogenesis is suggested by the increased incidence of MB in people with Li Fraumeni syndrome, which is caused by germ-line mutation in *p53* [28], [29], as well as in rodent models in which *p53* is co-deleted with genes such as *Ptch1* [30], [31]. P53 induces multiple anti-proliferative processes, including cell cycle arrest [32] and apoptosis [33], thereby effectively limiting the survival of potentially pre-neoplastic cells. Under physiological conditions, the level and activity of p53 is kept under strict negative control by the ubiquitin ligase, MDM2. Mice lacking *Mdm2* die *in utero* due to massive, p53-dependent apoptosis and this phenotype is fully rescued by deletion of p53 [34], [35]. In contrast, expression of a p53 mutant, p53R172P, that can stimulate growth arrest but not apoptosis, only partially rescues the *Mdm2* null phenotype, such that a subset of pups survives to postnatal day 12–15 [36], [37]. These mice exhibit a severe impairment in the proliferation of GNPs, resulting in shortened folia and diminished IGL cell numbers, strongly implicating MDM2 and p53 in cerebellar development. However, this study did not assess the influence of MDM2 on either p53-mediated apoptosis or Shh signaling during cerebellar development. Furthermore, the early death of these mice precluded an assessment of the consequences of MDM2 loss in tumorigenesis or behavior [36], [37].

Here, we took advantage of a unique hypomorphic *Mdm2* allele (*Mdm2^puro^*) to examine the contribution of this ubiquitin ligase to cerebellar development in the presence of its target, wild-type p53 [38]. This study reveals a threshold amount of MDM2 is required to inhibit the apoptotic function of p53 and facilitate Shh signaling in GNPs. Moreover, Shh signaling regulates the level of MDM2 in GNPs, suggesting a complex interplay between the p53 and Shh pathways in these important neuronal precursor cells. Together, these findings place MDM2 at a critical node between the p53 and Shh pathways in cerebellar development and tumorigenesis.

## Materials and Methods

### Ethics Statement

Mouse experiments were approved in advance by either the Purdue University Animal Care and Use Committee (protocol number 08-065; S.M.M.) or the NCI Frederick Animal Science Program (protocol number 08-006; M.E.P.) and performed in compliance with national regulatory standards.

### Mouse breeding and genotyping

Mouse strains used in this study have been described previously. *Mdm2^+/puro^* and *Mdm2^+/Δ7-9^* mice were maintained on a 129/Sv and C57BL/6 background, respectively, and interbred to obtain experimental, F1 hybrid *Mdm2^puro/Δ7-9^* and wild-type mice [38]. *P53^+/−^* heterozygotes on both 129/Sv and C57BL/6 backgrounds were obtained from Karlyne Reilly of NCI-Frederick [39]. B6;129-*Ptch1^tm1Mps^*/J (referred to as *Ptch1* ^+/−^) mice were purchased from The Jackson Laboratory (Bar Harbor, Maine) [18]. For analysis of pre-neoplastic lesions (PNLs), *Ptch1* ^+/−^;*Mdm2^+/puro^* and *Ptch1* ^+/−^;*Mdm2^+/Δ7-9^* mice were bred to obtain mice that express one of four levels of MDM2. Mice used for the PNL study were maintained on a mixed 129/Sv×C57BL/6 background. *p53*, *Ptch1* and *Mdm2* alleles were genotyped as described previously [38], [39], [40].

### Behavioral analysis

Eighteen wild-type and 17 mutant 4-month-old mice were assessed for gross neurological function, motor control, and balance using a series of well established behavioral tests [41]. To evaluate motor coordination and balance, mice were tested for the ability to stay on a cylindrical drum (Rotarod) that was either rotating at a constant speed (7 rpm) or accelerating (from 3.5 rpm to 35 rpm). In addition, 16 wild-type and 15 mutant 4-week-old mice were assessed on a subset of the tests and 8 wild-type and 5 mutant pups were assessed daily for the ability to right themselves immediately, starting at P7.

### Morphometic analysis

To determine the relative sizes of the white matter and IGL in adult cerebella, midsagittal sections from wild-type and *Mdm2^puro/Δ7-9^* mice were assessed. The areas of both layers were estimated using Image-Pro Plus v6.2 software (Media Cybernetics, Bethesda, MD). Images were acquired using a ScanScope CS (Aperio, Vista, CA) slide scanner at 40×. Areas of interest within the digital slides were extracted as TIFs at 100% resolution using ImageScope (Aperio, Vista, CA). The areas of the white matter and of the IGL were each determined by user-assisted region-of-interest selection and thresholding.

### Analysis of proliferation and apoptosis

For BrdU analysis of P0 pups, 95 mg BrdU/kg body weight was injected intraperitoneally one hour prior to necropsy. Brains were fixed for 24 hours in 10% neutral buffered formalin and embedded with paraffin and 5 micron sections were prepared. BrdU was detected immunohistochemically using antibody A21301MP (Invitrogen, Carlsbad, CA). TUNEL assay was performed using ApoTag Kit #S7100 (Millipore, Billerica, MA). To determine the percentage of BrdU-positive and TUNEL-positive EGL cells, the area of the EGL was estimated as described under morphometric analysis. The EGL was outlined manually using ImageScope software and the number of pixels representing hematoxylin-positive nuclei within that outline was determined using ImagePro software. The number of BrdU-positive EGL cells was estimated by automated determination of the number of pixels representing diaminobenzidine-positive nuclei as measured by signals equal to or greater than the average threshold range for the BrdU signal measured across several samples. Data were extracted as pixel area for both the EGL and BrdU signals. The percentage of BrdU-positive cells within the isolated EGL layer segmentation was determined by dividing the number of pixels representing BrdU-positive nuclei by the number of pixels representing EGL nuclei and multiplying by 100 for each sample. TUNEL-positive cells were counted under a microscope at 40× power. To calculate the percentage of EGL cells that was apoptotic, the EGL area was converted to cell number using the average EGL cell area (in pixels).

### Immunohistochemistry

Sagittal sections were stained with hematoxylin and eosin (H&E). Immunohistochemical detection of p53, Calbindin-D-28K, and CyclinD1 was performed using primary rabbit antibodies NCL-p53-CM5p, (Vector Laboratories, Burlingame, CA), KD-15 (Chemicon, Carpinteria, CA), and RB-212-P1 (Lab Vision/Thermo Fisher Scientific, Fremont, CA), respectively.

### Pre-neoplastic lesion (PNL) analysis

*Ptch1* ^+/−^ mice expressing one of four levels of MDM2 were euthanized at 3 weeks of age and cerebella were removed and fixed overnight in 4% paraformaldehyde and embedded in paraffin. The entire cerebellum of each animal was sagitally sectioned at 200 µm intervals. PNLs were identified morphologically in H&E stained sections as areas of focal or diffuse hyperplasia. Sections were analyzed and PNLs scored blindly and independently by 2–3 observers.

### Granular neuronal precursor (GNP) cell isolation

GNP cultures were prepared from pooled P5 cerebella of mice and stimulated with either 3 µg/ml recombinant Shh (20 kD amino-terminal signaling peptide; R&D Systems, Minneapolis, MN) or vehicle control as described by [15]. Shh stimulation of GNP proliferation was confirmed by adding 10 µM BrdU to the GNP culture media for 2 h followed by fixation in 70% ethanol. Proliferating cells that incorporated BrdU during the labeling period were identified using an anti-BrdU antibody and detected by immunofluorescent microscopy. The fold change in cell proliferation was calculated as the ratio of the percent BrdU-positive cells in cultures treated with Shh compared to vehicle only controls. At times post-Shh stimulation GNPs were washed twice in cold PBS and snap frozen in liquid nitrogen for subsequent RNA and protein analyses.

For the analysis of Shh and p53 target gene expression in GNPs that differ in the level of MDM2 expression, GNPs were isolated from individual cerebella and enriched by pre-plating on poly-D-lysine twice for 30-minutes. GNPs were subsequently quantified manually with a hemacytometer and snap frozen in liquid nitrogen. Significance between the number of GNPs isolated from mice differing in *Mdm2* genotype was determined using a two-tailed t-test.

### Western analyses

Immunoprecipitation-Western analyses of MDM2 in whole brain tissue was performed exactly as described in [38]. For Western analysis of proteins from GNPs, cell were washed twice in cold PBS and lysed in radioimmunoprecipitation assay (RIPA) buffer [42] supplemented with a protease inhibitor cocktail (Roche, Indianapolis, IN). Protein concentrations were determined using the DC Protein Assay (Bio-Rad, Hercules, CA). The primary antibodies for Western analyses were: anti-MDM2 (2A10; ab16895), anti-Gli (ab7523), anti-Gli2 (ab26056) (all from Abcam, Cambridge, MA) and anti-Actin (clone AC-15; Sigma, St. Louis, MO). Primary antibodies were revealed using the appropriate sheep anti-mouse or anti-rabbit secondary antibodies conjugated to peroxidase and enhanced chemiluminescence (ECL) (GE Healthcare, Piscataway, NJ).

### Real-time and RT-PCR

Total RNA isolated from cerebellar tissue was analyzed by reverse transcription (RT)- and real-time PCR. Whole cerebella were homogenized in Trizol (Invitrogen, Carlsbad, CA) and total RNA isolated per the manufacturer's instructions. Total RNA (2 µg) was used to generate cDNA using Transcriptor Reverse Transcriptase (Roche, Indianapolis, IN) and random hexamers. The following primers for mouse *Gli1*, *NMyc*, *CyclinD1*, *p21*, *DAPK*, *CyclinG*, *Mdm2*, and *Tubb5* control were designed to amplify products spanning gene introns with assistance of the Primer3 website: Gli1, 5′ GTG TAC CAC ATG ACT CTA CTC GGG 3′ and 5′ TCA TAC ACA GAC TCA GGC TCA GG 3′; *NMyc*, 5′ AGC ACC TCC GGA GAG GAT AC 3′ and 5′ AAG TGG TTA CCG CCT TGT TG 3′; *CyclinD1*, 5′ TCA AGA CGG AGG AGA CCT GT 3′ and 5′ CTC CTC TTC GCA CTT CTG CT 3′; *p21*, 5′ TTG TCG CTG TCT TGC ACT CT 3′ and 5′ TGC CAT CTG CGT CTA CCA GAC 3′; *DAPK*, 5′ TTG CAC AAC AGC TAC ACA GC 3′ and 5′ ATA GTC CCA CTA CTC AGG TC 3′; *CyclinG*, 5′ GCA TGG CAG CAC ATC CCT TTA 3′ and 5′ TGT AGA CCA GCC TGG CTT TGA AT 3′; *Mdm2*, 5′ AGC AGC GAG TCC ACA GAG A 3′ and 5′ ATC CTG ATC CAG GCA ATC AC 3′; and *Tubb5*, 5′ TGG GAC TAT GGA CTC CGT TC 3′and 5′ AAA GCC TTG CAG GCA ATC A 3′. Real-time and endpoint PCR were conducted to confirm amplification of a single product of the expected size. Real-time PCR reactions were set up using ABI SYBR green PCR Master Mix (ABI, Foster City, CA) and run on an ABI 7000 Sequence Detection System. Data were analyzed using ABI GeneAmp SDS software. Relative gene expression levels were calculated using the formula 2^−ΔΔCT^ with *Tubb5* as a loading control and wild-type expression values as the calibrator. Analyses were performed in triplicate for three mice per *Mdm2* genotype. One-way analysis of variance (ANOVA) test was done using SPSS 16.0 (SPSS Inc., Chicago, IL) with *P*-value<0.05 considered significant.

## Results

### The *Mdm2^puro^*allele is globally hypomorphic in expression

**Figure 1:**
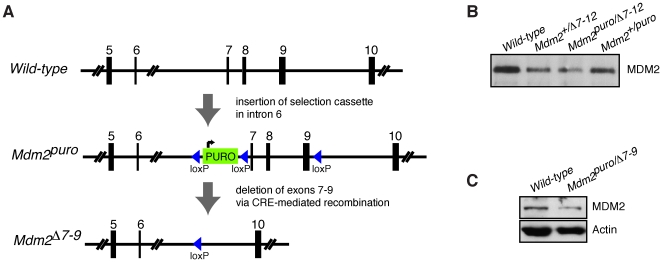
Generation and characterization of the hypomorphic *Mdm2^puro/Δ7-9^* allele. (A) Schematic illustrating partial structure of the *Mdm2* gene surrounding the site of gene targeting, the resulting *Mdm2^puro^* hypomorphic allele, and the *Mdm2^Δ7-9^* null allele generated following Cre-mediated recombination. (B) Immunoprecipitation followed by Western blot (WB) analysis of MDM2 in brains from mice of the indicated *Mdm2* genotype. (C) WB analysis of MDM2 in GNPs isolated from P5 mice of the indicated *Mdm2* genotype. WB for actin controls for loading.

During the production of a conditional *Mdm2* allele, we fortuitously generated a novel hypomorphic allele, *Mdm2^puro^* (Fig. 1A) [38]. Northern blot analyses of RNA derived from various tissues obtained from mice carrying one *Mdm2^puro^* allele and one null *Mdm2* allele (*Mdm2^Δ7-12^* [34] or *Mdm2^Δ7-9^* [38]; collectively referred to as *Mdm2^null^*) revealed that expression of full-length *Mdm2* mRNA was significantly reduced in all tissues examined including spleen, thymus, kidney, liver, and brain [38]. Western blot analysis of protein extracts prepared from *Mdm2^puro/null^* tissues confirmed reduced expression of full-length MDM2 protein in testes [38], thymus [43], and brain (Fig. 1). Consistent with the previously reported decrease in *Mdm2* mRNA in brain tissue, MDM2 protein levels are reduced three-fold in whole brain and four-fold in isolated cerebellar granular neuronal precursors (GNPs) (Fig. 1B,C). No aberrant *Mdm2* mRNAs or truncated MDM2 proteins have been identified from the *Mdm2^puro^* allele. The diminished expression of MDM2 from the *Mdm2^puro^* allele is dependent upon insertion of the *Puro* gene into the non-coding intron 6 of the *Mdm2* gene [38]. The mechanism by which the *Puro* gene diminishes *Mdm2* expression is not fully understood but may involve transcriptional interference as has been observed for other targeted alleles [44]. Together, these data establish that the *Mdm2^puro^* allele is globally hypomorphic for full-length wild-type MDM2 expression.

By combining wild-type, null, and hypomorphic alleles of *Mdm2* we are able to generate mice that differ in the level of MDM2 expression (Fig. 1). Importantly, a 70% decrease in the level of MDM2 is sufficient for survival but results in multiple overt p53-dependent phenotypes in *Mdm2^puro/null^* mice, including reduced body size and lymphopenia that are not manifest in mice with a more modest decrease in MDM2 [38]. *Mdm2^puro/Δ7-9^* mice thus provide a unique model for perturbing MDM2 and p53 function in the developing cerebellum in order to assess their impact on Shh signaling.

### Hypoplasia and aberrant foliation in adult *Mdm2^puro/Δ7-9^* cerebella

**Figure 2:**
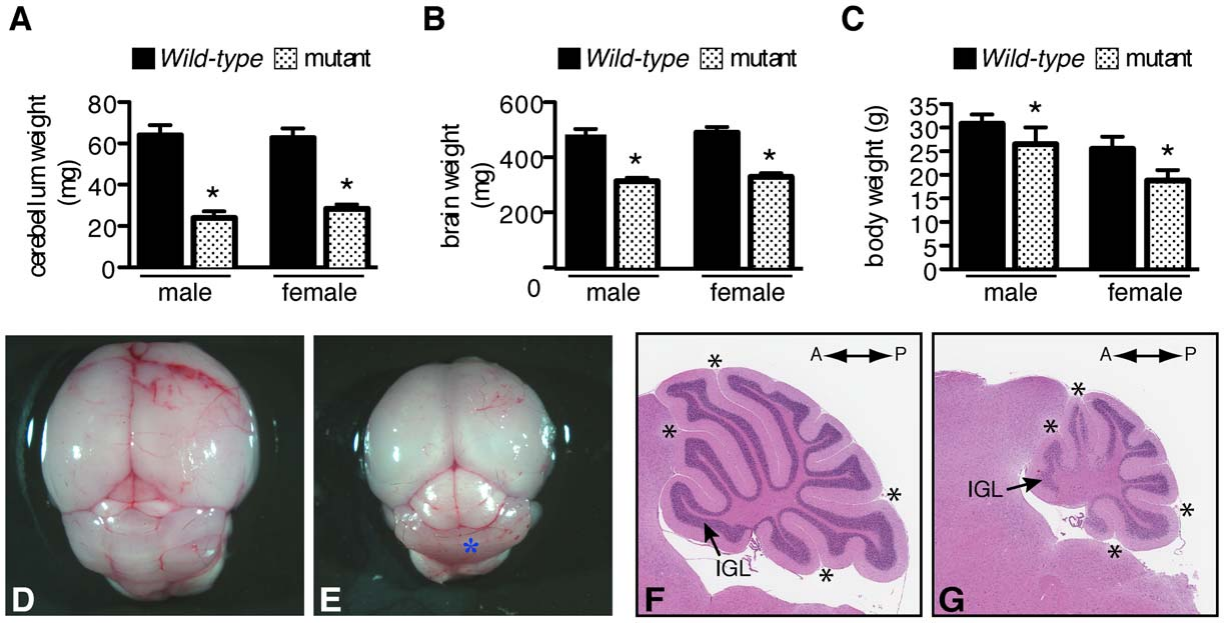
Cerebellar size and foliation are defective in *Mdm2^puro/Δ7-9^* mice. In 4-month-old male and female *Mdm2^puro/Δ7-9^* mice, cerebellar weight (A) is disproportionately reduced from wild-type relative to the decreases in both brain weight (B) and body weight (C) (*n* = 5–10, *P*<0.001 when comparing wild-type and mutant mice by sex). Gross analysis of whole brains of 1-month-old wild-type (D) and *Mdm2^puro/Δ7-9^* mice (E). The cerebellar vermis in mutant mice, denoted by the asterisk in (E), is reduced in mass thereby exposing more of the colliculi. H&E stained midsagittal sections of 4-month-old wild-type (F) and *Mdm2^puro/Δ7-9^* (G) cerebella. *Mdm2^puro/Δ7-9^* cerebella show a marked agenesis of foliation, with the internal granular layer (IGL) more severely diminished at the anterior and posterior folia. The asterisks in (F) and (G) denote the principal fissures which are shallow in mutant mice.

Macroscopic examination of brains from adult *Mdm2^puro/Δ7-9^* mice revealed cerebella that were strikingly reduced in size. At 4 months of age, both male and female *Mdm2^puro/Δ7-9^* mice showed a greater than two-fold reduction in cerebellar mass (Fig. 2A). This decrease was more profound than the decrease in overall brain weight (33% for males and 35% for females; Fig. 2B) or body weight (16% for males and 23% for females; Fig. 2C), indicating that the cerebellum is especially sensitive to a decrease in the level of MDM2. Gross inspection of adult brains suggested a marked agenesis of the cerebellar vermis (Fig. 2D,E), which was corroborated by histological analysis (Fig. 2F,G). Midsagittal sections of 4-month old *Mdm2^puro/Δ7-9^* mice showed reduced foliation associated with an IGL that was thin or absent, particularly at the base of the fissures, suggesting a reduction in the number of mature neurons. Morphometric analysis revealed that the total area of the cerebella of *Mdm2^puro/Δ7-9^* mice was reduced by 69% while the area of the IGL was reduced by 76% and the area of the white matter was reduced by 56% (*n* = 8–9, *P*<10^−6^ for all three parameters). Importantly, sparseness of the IGL is most apparent in the most anterior and posterior cerebellar lobes, corresponding to regions of the highest Shh signaling [45]. However, abnormalities in foliation are not restricted to any one area, as all four principal fissures (preculminate, primary, secondary and posterolateral) are shallow. In addition to the principal fissures, only three of several other shallow fissures are present, similar to the phenotype of mice in which the Shh effector Gli2 has been deleted either in the germ line or conditionally in the embryo [45]. Foliation is markedly sensitive to the level of MDM2 as we found no overt abnormalities in *Mdm2^+/Δ7-9^* cerebella in which the level of MDM2 is ∼50% of wild-type (data not shown).

In light of the observed cerebellar hypoplasia of *Mdm2^puro/Δ7-9^* mice, a number of tests were performed to assess gross neurological function, motor coordination, and behavior (see Table S1 for details). Despite the severe structural defects of the cerebellum, *Mdm2^puro/Δ7-9^* mice were, for the most part, functionally indistinguishable from wild-type mice. Only one significant difference was noted: *Mdm2^puro/Δ7-9^* mice remained 1.5 times longer on an accelerating, rotating drum (Rotarod; *n* = 17–18, *P*<0.001). This difference in behavior is consistent with a lack of profound defects in motor coordination, suggesting that *Mdm2^puro/Δ7-9^* mice are able to compensate for the severe structural changes in their cerebella.

### Delayed formation of fissures in *Mdm2^puro/Δ7-9^* cerebella

**Figure 3:**
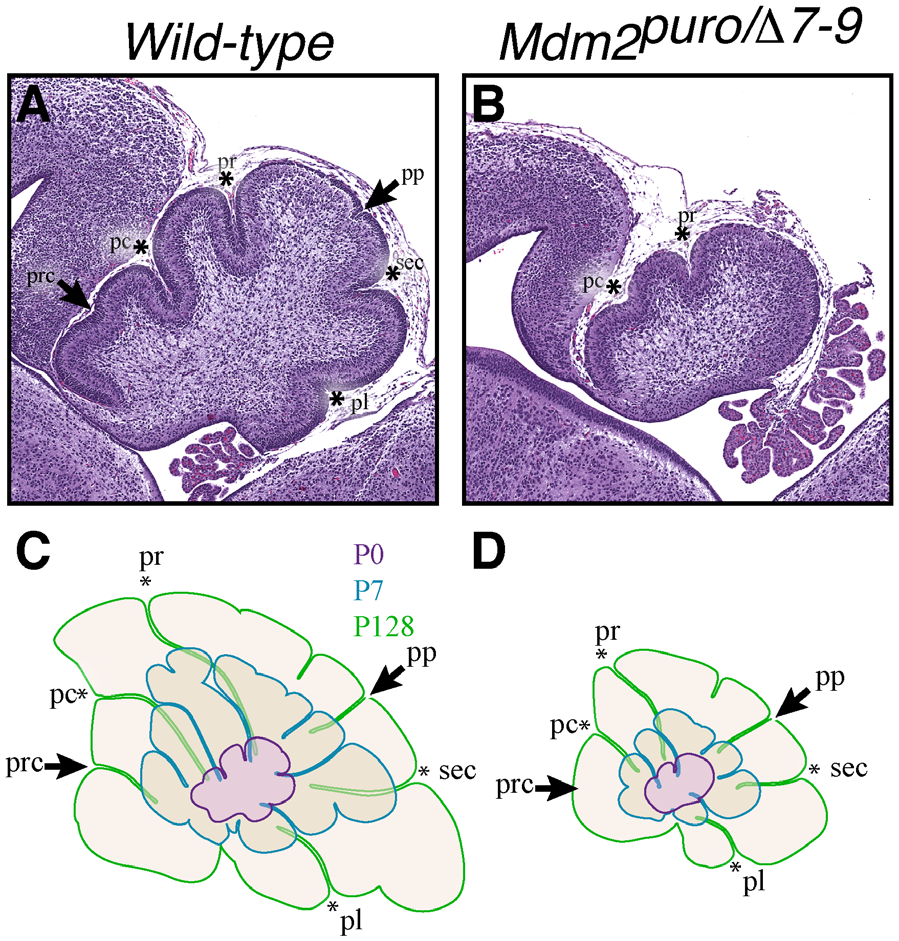
Foliation defects in P0 mice. (A–B) Midsagittal sections of newborn (P0) wild-type and *Mdm2^puro/Δ7-9^* cerebella stained with H&E. The four principal fissures, denoted by asterisks, are apparent in wild-type cerebella at P0 (A), whereas only two are evident in *Mdm2^puro/Δ7-9^* mice (B). Wild-type pups show two additional fissures, indicated by arrows, whereas mutant pups do not. (C–D) Superimposition of P0 (purple outline), P7 (blue outline), and adult (green outline) cerebella from wild-type (C) or *Mdm2^puro/Δ7-9^* (D) mice. By P7, all four primary fissures, as well as two additional fissures, are evident in *Mdm2^puro/Δ7-9^* mice. Moreover, even in adulthood, the mutant cerebellum does not reach the size or complexity of the wild-type cerebellum. Abbreviations are: prc, precentral; pc, pre-culminate; pr, primary; pp, prepyramidal; sec, secondary; pl, posterolateral fissures.

Post-natal expansion of the EGL continues for approximately three weeks, and is necessary for proper foliation. Previous studies have shown the cerebellar surface of outbred Swiss Webster embryos is smooth at E16.5, but develops four principal fissures and five cardinal lobes by E18.5 [6]. Two additional fissures form by P3, subdividing both the anterobasal lobe and lobule VI. Foliation appears to occur slightly earlier in wild-type F1 hybrid 129Sv/C57BL6 mice as by P0, the four principal fissures have formed, as have two additional fissures separating the anterobasal lobe and lobe VI (Fig. 3A). In contrast, P0 *Mdm2^puro/Δ7-9^* mice have only two primary fissures (Fig. 3B). Although preculminate and primary fissures were apparent in all *Mdm2^puro/Δ7-9^* mice at P0, the secondary and posterolateral fissures were not often visible. Moreover, all fissures were shallow in newborn *Mdm2^puro/Δ7-9^* mice. Foliation depends on thickening of the EGL layer at specific anchor points [6], one or more of which appears to be missing in P0 *Mdm2^puro/Δ7-9^* mice. Indeed, the number of EGL cells was reduced three-fold in P0 *Mdm2^puro/Δ7-9^* mice (*P*<0.001), indicating that an insufficient number of GNPs may be available to form the anchor points and to extend the lobules. Superimposition of traces of mid-sagittal sections from P0, P7, and adult mice revealed that the appearance of the secondary and posterolateral fissures was delayed in *Mdm2^puro/Δ7-9^* mice and that all fissures remained shallow throughout development (Fig. 3C,D). The shallow principal fissures at birth (P0) and delayed lobularization of *Mdm2^puro/Δ7-9^* mice are reminiscent of the foliation defects observed in mice with decreased Shh signaling [45].

### Impaired expansion of the EGL in *Mdm2^puro/Δ7-9^* mice

**Figure 4:**
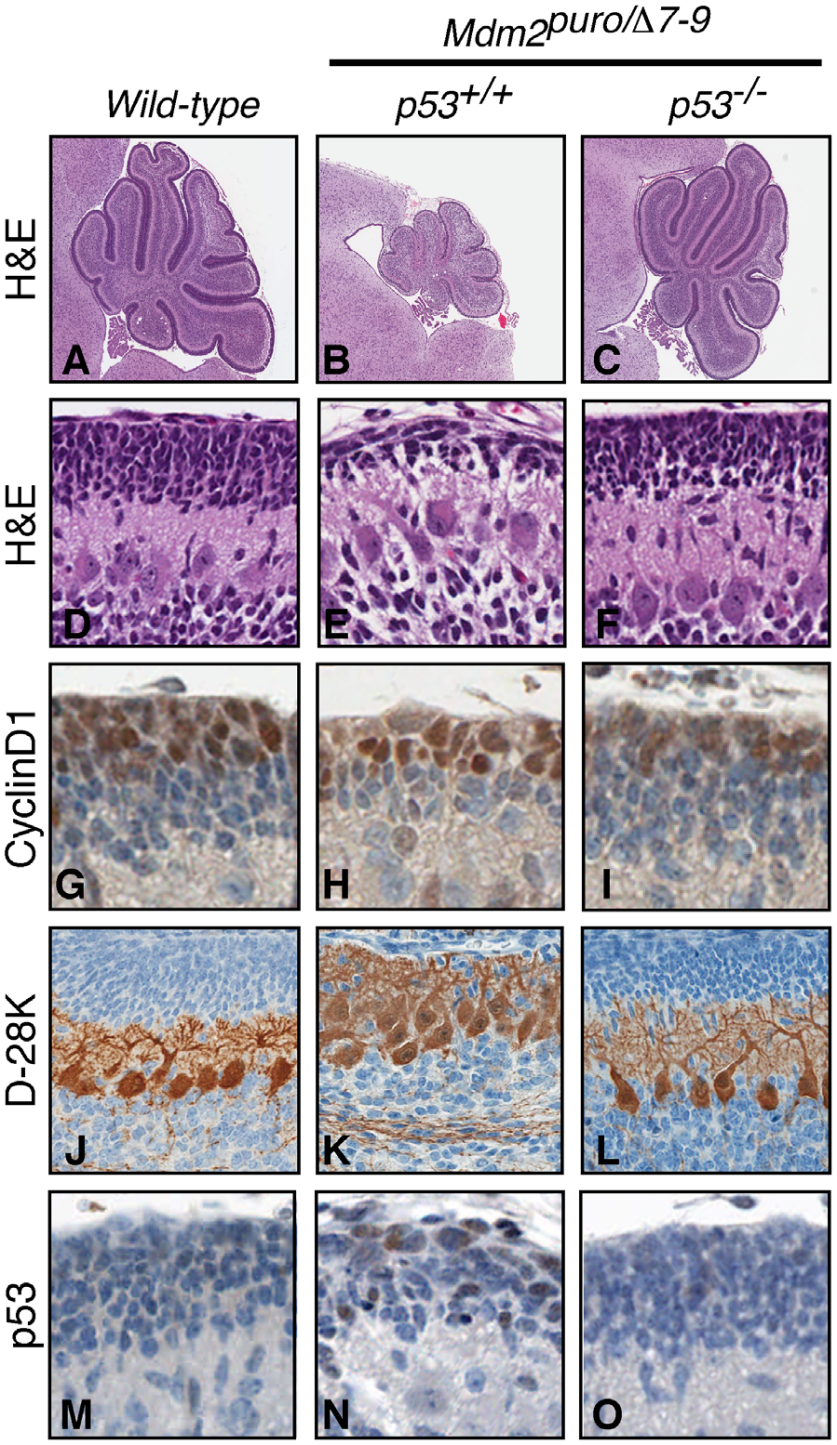
Cerebellar abnormalities of *Mdm2^puro/Δ7-9^* mice are rescued by loss of p53. Midsagittal sections of cerebella from wild-type (A,D,G,J,M), *Mdm2^puro/Δ7-9^* (B,E,H,K,N), and *Mdm2^puro/Δ7-9^;p53^−/−^* (C,F,I,L,O) mice at P7. (A–F) H&E staining shows a thin EGL and sparsely-populated IGL in *Mdm2^puro/Δ7-9^* cerebella. (G–I) MDM2 deficiency does not appear to block GNP differentiation as immunohistochemical staining for CyclinD1 selectively marks undifferentiated GNPs of the outer, but not inner, EGL. (J–L) *Mdm2^puro/Δ7-9^* Purkinje cells stained with anti-Calbindin D-28K have stunted arborization of the dendrites and have failed to form a uniform monolayer as seen in wild-type controls. *Mdm2^puro/Δ7-9^;p53^−/−^* (M–O) Immunohistochemical staining for p53 reveals an increase in the number of GNPs staining positively for p53 in the EGL of *Mdm2^puro/Δ7-9^* mice. (C,F,I,L,O) *Mdm2^puro/Δ7-9^;p53^−/−^* mice are phenotypically indistinguishable from wild-type mice, highlighting the importance of MDM2 for limiting p53 function in cerebellar development.

By P7, the EGL of wild-type mice has reached its maximal thickness and the IGL has begun to form as GNPs differentiate and migrate inward past the Purkinje cells. Cerebella from *Mdm2^puro/Δ7-9^* mice had a thinner EGL than that of wild-type mice, which had expanded to an 8–10 cell thick layer (Fig. 4A,B,D,E). *Mdm2^puro/Δ7-9^* EGL cells appeared capable of differentiating, as the inner EGL of both wild-type and *Mdm2^puro/Δ7-9^* mice was negative for CyclinD1, a marker of undifferentiated GNPs (Fig. 4G,H). Immunostaining for Calbindin-D-28K revealed that, by P7, the Purkinje cells in wild-type mice had adopted a uniform monolayer with dense dendritic arborization (Fig. 4J). However, in *Mdm2^puro/Δ7-9^* cerebella, the Purkinje cells remained multi-layered and disorganized with stunted dendritic arborization (Fig. 4K). As the position of Purkinje cells within the cerebellum is informed by the expanding folia, these results provide additional support for a diminished EGL cell number as the underlying basis for the observed defects in foliation in *Mdm2^puro/Δ7-9^* cerebella.

### P53-dependence of cerebellar defects of *Mdm2^puro/Δ7-9^* mice

Consistent with the reduction in the level of the MDM2 ubiquitin ligase in *Mdm2^puro/Δ7-9^* cerebella, the level of p53 protein was elevated, as revealed by the increased percentage of EGL cells immunoreactive for p53 (Fig. 4N). To establish whether increased p53 activity was responsible for the diminished granular layer cell numbers and foliation defects of *Mdm2^puro/Δ7-9^* mice, cerebella from *Mdm2^puro/Δ7-9^* mice lacking p53 were assessed. *Mdm2^puro/Δ7-9^;p53^−/−^* and wild-type cerebella were indistinguishable in size and morphology (Fig. 4A,C). Histological analysis showed that in *Mdm2^puro/Δ7-9^;p53^−/−^* mice, foliation was normal, as was the thickness of both the EGL and IGL (Fig.4,C,D,F). Morphometric analysis of the EGL revealed that *Mdm2^puro/Δ7-9^* mice had a 73% decrease in the number of EGL cells (*P*<10^−5^) whereas *Mdm2^puro/Δ7-9^;p53^−/−^* mice had an insignificant (3%) increase when compared to wild-type mice (*P* = 0.40). Similarly, the number and organization of the Purkinje cells was rescued in *Mdm2^puro/Δ7-9^;p53^−/−^* mice (Fig. 4K,L). Whereas the number of Purkinje cells was reduced 33% in *Mdm2^puro/Δ7-9^* mice (*P* = 0.00014), it was insignificantly reduced in *Mdm2^puro/Δ7-9^;p53^−/−^* mice (11%; *P* = 0.14 compared to wild-type). Together, these results indicate that MDM2 protects the processes of GNP expansion and cerebellar foliation by inhibiting p53.

### Decreased survival of *Mdm2^puro/Δ7-9^* granule neuron precursors

**Figure 5:**
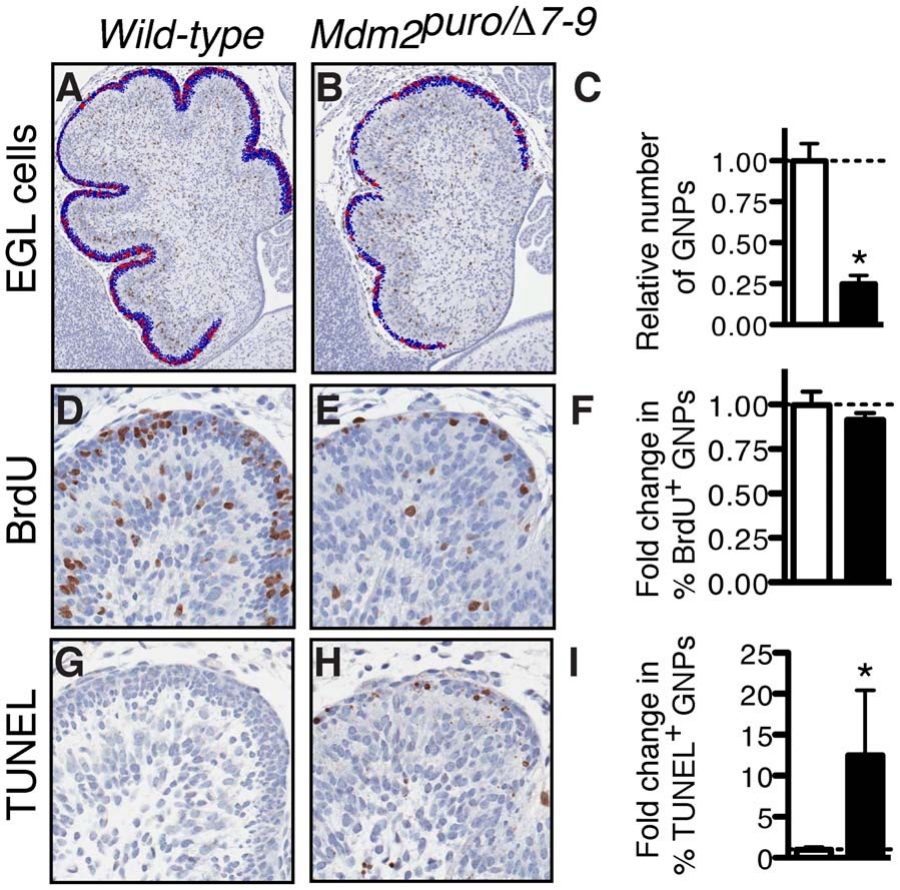
Decreased proliferation and increased apoptosis in *Mdm2^puro/Δ7-9^* cerebella. (A,B) Identification of BrdU-positive (red) cells in the EGL (blue) of midsagittal sections of newborn (P0) wild-type (A) and *Mdm2^puro/Δ7-9^* (B) cerebella using ImagePro software as described under Materials and Methods. The EGL of *Mdm2^puro/Δ7-9^* cerebella was acellular (blue) and hypoproliferative (red) as revealed by BrdU staining followed by image analysis. (C) Graph of the relative number of EGL cells in wild-type and *Mdm2^puro/Δ7-9^* cerebella (*n* = 3, *P*<0.001). (D,E) High magnification of BrdU label in tip of lobule III in a wild-type (D) and an *Mdm2^puro/Δ7-^* ^***9***^ (E) cerebellum. (F) Graph of relative percentage of BrdU-positive cells in EGL of wild-type and *Mdm2^puro/Δ7-9^* cerebella (*n* = 3, *P* = 0.082). (G,H) High magnification of TUNEL label in end of lobule III in a wild-type (G) and an *Mdm2^puro/Δ7-9^* (H) cerebellum. (I) Graph of relative percentage of TUNEL-positive cells in EGL of wild-type and *Mdm2^puro/Δ7-^* ^***9***^ cerebella. GNPs in the EGL of *Mdm2^puro/Δ7-9^* cerebella are highly apoptotic (*n* = 3, *P* = 0.033).

Two physiological outcomes associated with heightened p53 activity are growth arrest and apoptotic cell death, both of which could contribute to a decrease in the number of GNPs. To investigate whether a reduction in cell proliferation contributes to the diminished GNP population in P0 *Mdm2^puro/Δ7-9^* mice, we performed *in vivo* labeling with 5-bromo-2-deoxyuridine (BrdU). We found that the EGL of both wild-type and *Mdm2^puro/Δ7-9^* mice was proliferative, but that there were fewer BrdU-positive GNPs in the MDM2-deficient cerebella. The absolute number of proliferating BrdU-positive cells in the EGL in P0 *Mdm2^puro/Δ7-9^* cerebella was only 23% of that found in wild-type controls (Fig. 5A,B,D,E). However, when a four-fold decrease in the number of GNPs in P0 *Mdm2^puro/Δ7-9^* mice (Fig. 5C) was taken into account, the relative number of BrdU-positive GNPs was 91% that in wild-type controls (Fig. 5F). The apparent reduction in GNP proliferation in the *Mdm2^puro/Δ7-9^* EGL is therefore predominantly attributed to a paucity of GNPs rather than a decrease in the fraction of progenitor cells in S phase. Although the decrease in the number of BrdU-positive *Mdm2^puro/Δ7-9^* GNPs was statistically insignificant (*P* = 0.082) as compared to wild-type, GNPs proliferate at a high rate during the first week of post-natal cerebellar development. Thus, even a modest decrease in proliferation could significantly reduce the total number of GNPs generated and hence negatively impact cerebellar size and foliation.

As MDM2 also limits the apoptotic function of p53, we next assessed whether in *Mdm2^puro/Δ7-9^* mice GNP survival was impaired. To identify apoptotic cells, we performed terminal deoxynucleotidyl transferase dUTP nick end labeling (TUNEL) in situ. Quantitative analysis of cell death revealed an increase in the number of TUNEL-positive cells in the EGL of *Mdm2^puro/Δ7-9^* cerebella (Fig. 5G,H). In contrast to the moderate decrease in cell proliferation, the percentage of TUNEL-positive EGL cells was increased 12.4-fold in the *Mdm2^puro/Δ7-9^* cerebellum at P0 (*P* = 0.033; Fig. 5I). This increase in apoptosis persisted for at least seven days, as *Mdm2^puro/Δ7-9^* mice had a 20-fold increase in the fraction of EGL cells that were apoptotic at P7 (*n* = 3, *P*<0.035; data not shown). Consistent with the normal size and foliation of *Mdm2^puro/Δ7-9^;p53^−/−^* cerebella, the increase in apoptosis in the *Mdm2^puro/Δ7-9^* EGL is p53-dependent, as demonstrated by the normal (e.g., equivalent to wild-type) fraction of GNPs that were TUNEL-positive in the EGL of P7 *Mdm2^puro/Δ7-9^*mice lacking *p53* (*n* = 3, *P* = 0.39 when compared to wild-type; data not shown). Together, these data indicate that increased apoptosis rather than growth arrest is the main contributor to the diminished EGL in *Mdm2^puro/Δ7-9^* mice.

### Attenuated Sonic Hedgehog signaling in *Mdm2^puro/Δ7-9^* cerebella

**Figure 6:**
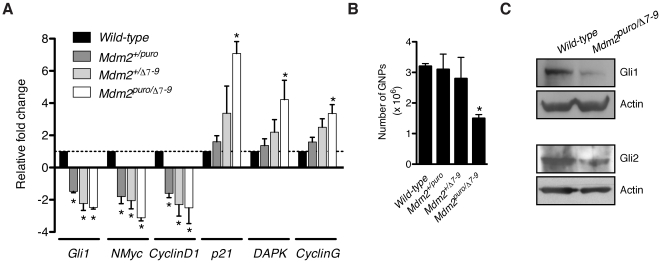
Shh signaling is attenuated concomitant with p53 activation in *Mdm2^puro/Δ7-9^*cerebella. (A) Expression of Shh target genes (*Gli1*, *NMyc*, *CyclinD1*) and p53 target genes (*p21*, *DAPK*, *CyclinG*) in whole cerebella was determined by real-time PCR and normalized to *Tubb5*. Graphed is the average fold change in gene expression of Shh- and p53-target genes in whole cerebella of P5 mice with the indicated *Mdm2* genotype relative to wild-type controls (*n* = 3, *P<0.05). (B) Histogram showing the total number of GNPs isolated from cerebella of mice of the indicated *Mdm2* genotype at P5 (*n* = 3–8, * *P*<0.05). (C) WB analysis of Gli1 and Gli2 in GNPs isolated from P5 mice of the indicated *Mdm2* genotype. WB for actin confirms equal loading.

As noted in the previous sections, we observed a striking similarity between the cerebellar phenotypes of *Mdm2^puro/Δ7-9^* mice and those lacking Shh [11] or its downstream effectors (e.g. Gli2, NMyc, CyclinD2) [45], [46], [47]. For example, the defects in EGL expansion, Purkinje cell layer organization and foliation are similar to those in which Gli2 has been deleted conditionally [45]. These observations prompted us to examine if Shh signaling was altered in the cerebellum of *Mdm2^puro/Δ7-9^* mice. We therefore measured the level of expression of known targets of Shh-activated transcription factors. Quantitative real-time PCR analyses revealed that expression of each of the direct Shh target genes *Gli1* and *NMyc* [15], as well as the indirect Shh target *CyclinD1* [48] was reduced 2- to 3-fold in P5 *Mdm2^puro/Δ7-9^* cerebella compared to wild-type control cerebella (Fig. 6A). Analysis of Shh signaling in an allelic series of *Mdm2-*deficient mice revealed that even an ∼20% reduction in MDM2 expression in *Mdm2^+/puro^* mice was sufficient to reduce Shh target gene expression (Fig. 6A). As the number of GNPs present in *Mdm2^puro/+^* mice is equivalent to that in wild-type mice (Fig. 6B), the reduction in Shh target gene expression in these mice cannot be explained by an under-representation of GNPs. Moreover, in the same samples, p53 target gene expression was increased as expected. The finding of reduced Shh target gene expression is consistent with a recent model in which enhanced p53 activity down-regulates Shh signaling [49].

### Gli1 and Gli2 proteins are decreased in *Mdm2^puro/Δ7-9^* GNPs

The Gli proteins are critical mediators of the Shh signal. To begin to establish the underlying basis by which a low level of MDM2 limits the output of Shh signaling in *Mdm2^puro/Δ7-9^* cerebellum, we analyzed the level of Gli1 and Gli2 in *Mdm2^puro/Δ7-9^* GNPs. Consistent with the reduction of *Gli1* mRNA in the *Mdm2^puro/Δ7-9^* cerebellum (Fig. 6A), the steady-state level of endogenous Gli1 is reduced seven-fold in *Mdm2^puro/Δ7-9^* GNPs as compared to wild-type GNPs (Fig. 6C). Significantly, the steady-state level of endogenous Gli2 protein was reduced two-fold in GNPs in which MDM2 is expressed at only ∼25% the level of wild-type (Fig. 6C). Our finding that Gli1 and Gli2 expression is reduced in *Mdm2^puro/Δ7-9^* GNPs in which p53 function is heightened suggests a model in which p53 attenuates Shh signaling output by negatively regulating Gli activity.

### Shh signaling induces MDM2

**Figure 7:**
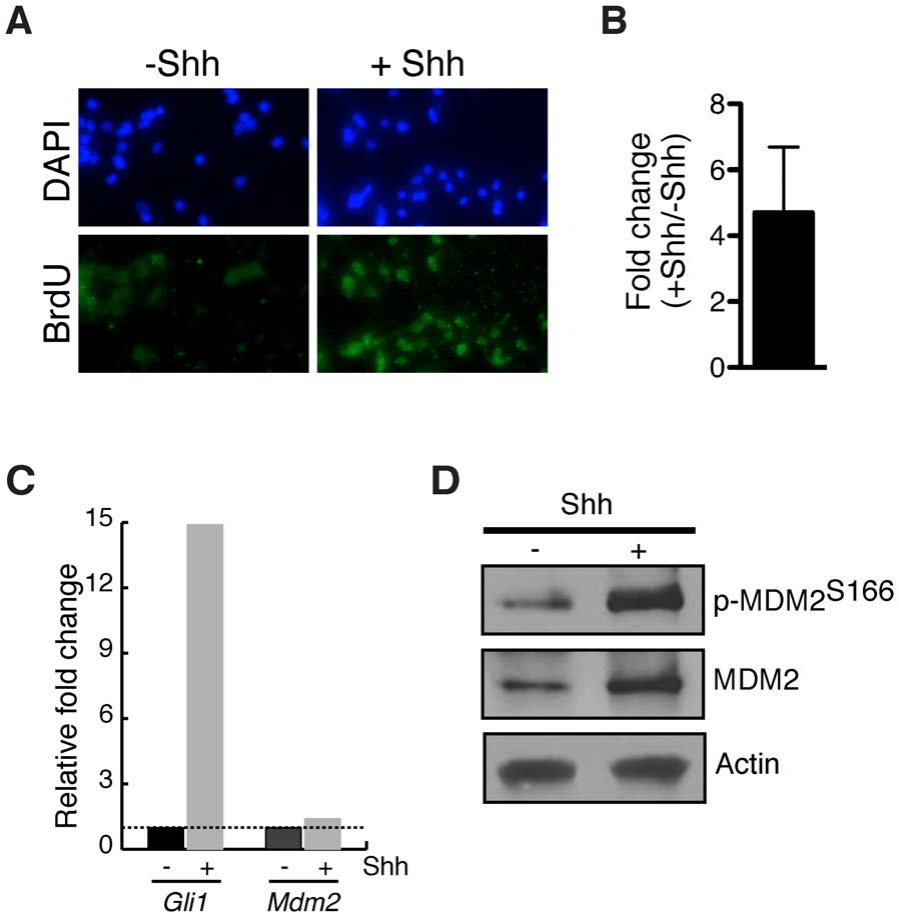
Regulation of MDM2 by Shh in cultured wild-type GNPs. (A) IHC for BrdU-incorporating cells in vehicle-treated (−Shh) and stimulated (+Shh) GNPs. Total nuclei labeled by DAPI. (B) Graphed is the average fold change in the percentage of GNPs immunoreactive for BrdU in Shh stimulated versus vehicle control cultures (n = 3). (C) Expression of *Mdm2* and the Shh target gene, *Gli1*, was quantified by real-time PCR and normalized to *Tubb5*. Graphed is the relative fold change in expression of *Mdm2* and *Gli1* in wild-type GNPs cultured in the presence of Shh (+) or vehicle (−). (B) WB analysis of MDM2 and p-MDM2^S166^ in GNPs treated with Shh (+) or vehicle (−). WB for actin confirms equal loading of protein.

Activation of the Shh pathway in C3H10T1/2 mesenchymal and mouse embryonic fibroblast (MEF) promotes phosphorylation of MDM2 at serine 166 (p-MDM2^S166^), a modification that enhances the ability of MDM2 to bind to and limit p53 function [50], [51], [52]. To determine whether MDM2 is similarly regulated by Shh signaling in GNPs, we employed primary cultures from cerebellar homogenates. Derived from mice at P5, >85% of the cells in cerebellar homogenates are GNPs [15], [48]. In our hands, <1.5% of cells in cultured from wild-type mice were GFAP^+^ glial cells (*n* = 2, data not shown). Addition of recombinant Shh-N to the culture medium increased proliferation ∼4.9-fold based on BrdU incorporation (Fig. 7A,B), consistent with other studies [15]. Concomitant with enhanced GNP proliferation, expression of the Shh-target *Gli1*was increased greater than 10-fold (Fig. 7C). Cultured GNPs thereby retain their *in vivo* characteristics and thus provide a good experimental system with which to dissect the molecular pathways that control their behavior. Using this experimental system, we discovered that both total MDM2 and its activated form, p-MDM2^S166^, accumulated 3- to 4-fold in GNPs stimulated by Shh, without a corresponding increase in *Mdm2* mRNA (Fig. 7C,D).

### Decreased incidence of pre-neoplastic lesions in *Ptch1^+/−^;Mdm2^puro/Δ7-9^* mice

**Figure 8:**
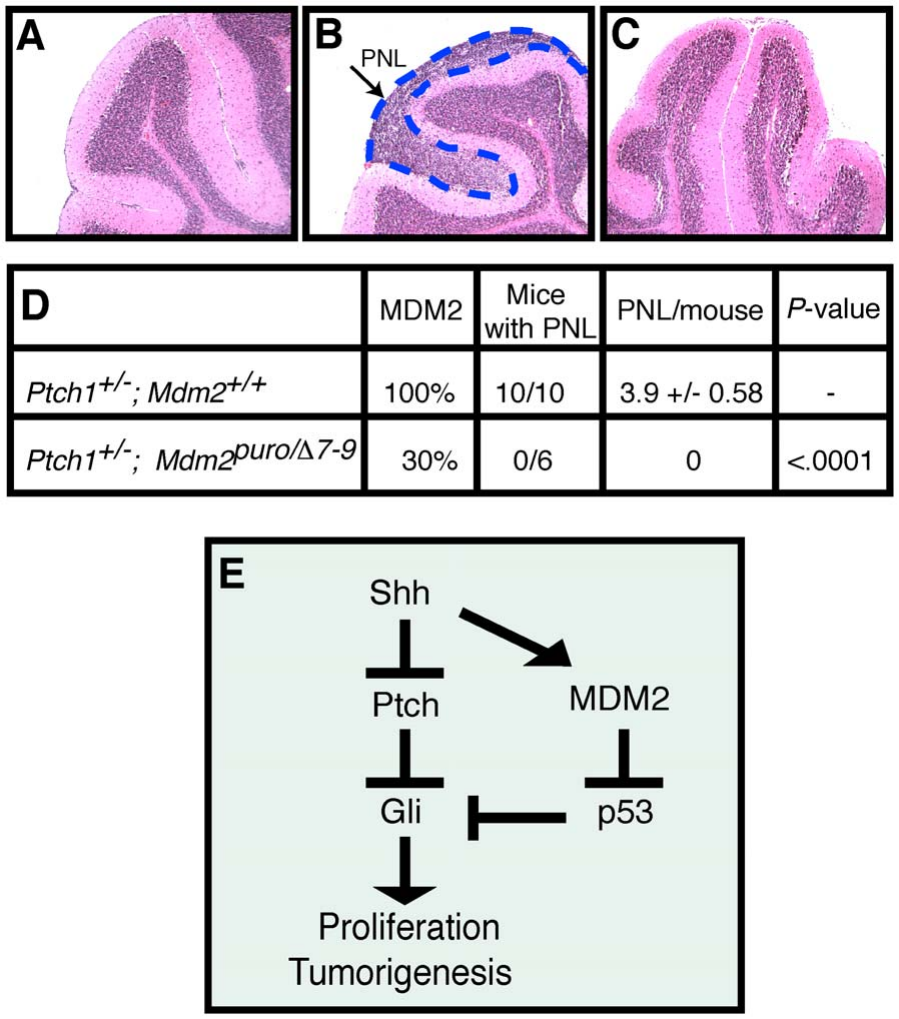
Shh-induced pre-neoplastic lesion (PNL) formation in the cerebellum is significantly decreased in *Ptch1^+/−^* mice expressing ∼30% the wild-type level of MDM2. (A–C) Shown are H&E stained sagittal sections of cerebella obtained from 3-week-old (A) wild-type, (B) *Ptch1^+/−^*, and (C) *Ptch1^+/−^*;*Mdm2^puro/Δ7-9^* mice. PNLs as indicated by the arrow and outlined by the dashed blue line in (B) are composed of small, round tightly clustered cells with a high nucleus∶cytoplasmic ratio located superficial to the molecular layer either on the surface of the cerebellum or in a fissure between adjacent folia. *Ptch1^+/−^*;*Mdm2^puro/Δ7-9^* mice (C) fail to develop PNLs. (*D*) Quantification of PNLs observed in 3-week-old *Ptch1^+/−^* and *Ptch1^+/−^*;*Mdm2^puro/Δ7-9^* mice. (E) Model for crosstalk between p53 and Shh pathways in cerebellar development. During GNP development, Shh stimulates proliferation through the positive regulation of the Gli family of transcription factors. P53 promotes apoptosis and concomitantly interferes with Gli function, attenuating the proliferative effects of Shh. Shh regulates MDM2, to keep p53 activity in check, allowing GNPs to expand in response to Shh signaling.

Deregulation of the Shh pathway is implicated in >60% of MB [23]. Based on our finding that MDM2 promotes Shh signaling as well as GNP survival during cerebellar development, we hypothesized that MDM2 also plays a critical role in Shh-induced cerebellar disease. As a test of this hypothesis, we performed a genetic loss-of-function study using *Ptch1^+/−^* mice, a model of Shh-induced human MB [18]. While only a subset of *Ptch1^+/−^* mice develop MB, close to 100% of *Ptch1^+/−^* mice exhibit clusters of highly proliferative cells at the surface of the cerebellum at 3 weeks of age, by which time the majority of GNPs in wild-type mice have stopped dividing, differentiated and migrated into the IGL as mature GCs [18], [40], [53]. Orthotopic transplantation and fate mapping experiments support the identity of these proliferating cells as uncommitted pre-neoplastic cells that are susceptible to transformation [54]. These clusters of proliferative cells therefore represent pre-neoplastic lesions (PNLs) for MB in this mouse model. To determine the requirement for MDM2 in the formation of PNLs, we examined cerebella of 3-week-old *Ptch1^+/−^* mice that differed in the level of MDM2. In agreement with prior studies, no PNLs were present in the cerebellum of wild-type mice (*n* = 5), whereas 100% of *Ptch1^+/−^* mice with a wild-type level of MDM2 (*n* = 10) exhibited multiple PNLs in the cerebellum (Fig. 8). Strikingly, despite analysis of six cerebella from *Ptch1^+/^;Mdm2^puro/Δ7-9^* mice, we have yet to identify any with a PNL. These results indicate that an approximately 70% reduction in *Mdm2* expression completely abrogates formation of Shh-induced PNLs in *Ptch1^+/−^* mice (Fig. 8A–D). These results suggest that MDM2 plays a critical role in GNPs during Shh-mediated MB formation.

## Discussion

Here we have taken advantage of a unique series of *Mdm2* alleles to obtain a comprehensive understanding of MDM2 function in the cerebellum. Whereas *Mdm2*-null mice die early in embryogenesis [34], [35], *Mdm2^puro/Δ7-9^* compound heterozygotes, which express only ∼30% of the wild-type level of MDM2 are viable but display multiple cerebellar defects including decreased expansion of the number of GNPs within the EGL, disorganization of the Purkinje cell layer, and aberrant foliation. In contrast, ∼50% expression of MDM2, as in *Mdm2^+/Δ7-9^* mice, is sufficient for cerebellar development to proceed normally, thus illustrating the sensitivity of this process to the level of MDM2. Consistent with the well-established role of MDM2 as a negative regulator of p53 function, cerebellar defects in *Mdm2^puro/Δ7-9^* mice correlate with an increase in p53 protein expression and an elevated rate of p53-mediated apoptosis in GNPs in the EGL suggesting that p53 is responsible for the dearth of granule cells in adult cerebella. This interpretation is borne out by the complete rescue of all phenotypes of *Mdm2^puro/Δ7-9^* mice by deletion of p53, and establishes a critical role for MDM2 in negatively regulating wild-type p53 function during normal cerebellar development. As EGL expansion is a driving force of foliation [6], the inability of the EGL to expand appropriately, due to the restricted number of GNPs in the *Mdm2^puro/Δ7-9^* cerebellum, likely underlies the foliation defects observed in these mice. These results implicate MDM2 as a major determinant of GNP cell survival and the extent of cerebellar foliation.

There is extensive crosstalk between GNPs and Purkinje cells in the developing cerebellum. Expansion of GNPs of the EGL is instructive for localization and differentiation of Purkinje cells [45]. Purkinje cells secrete Shh, which binds to and blocks the inhibitory activity of the Ptch1 receptor expressed on GNPs, thereby driving GNP proliferation and EGL expansion [11], [55]. Therefore, the failure of the EGL to expand in MDM2-deficient mice could either be due to a defect in GNPs or be secondary to a defect in Purkinje cells. While our work does not preclude a role for MDM2 in Purkinje cells, our results are consistent with an intrinsic requirement for MDM2 in GNPs. Several lines of evidence support this interpretation. (i) EGL expansion and foliation defects in *Mdm2^puro/Δ7-9^* cerebella coincide with p53-mediated apoptosis that is increased in GNPs but not in Purkinje cells. (ii) p53 expression is increased specifically in GNPs. (iii) There are approximately twice as many Purkinje cells per EGL cell in *Mdm2^puro/Δ7-9^* mice as in wild-type mice (*P* = 0.002). (iv) Reduced expression of Gli1 and Gli2, important transducers of the Shh signal, in GNPs supports a cell autonomous disruption in Shh signaling. Thus, the Purkinje cell phenotype of *Mdm2^puro/Δ7-9^* mice is likely a consequence of the paucity of GNPs.

In cerebellar development, MDM2 deficiency mimics aspects of Shh deficiency. The decreased number of granule cells and shortened length of the folia of *Mdm2^puro/Δ7-9^* cerebella are reminiscent of the phenotypes of cerebella from mice with reduced Shh signaling. *Shh*-null mice lack cerebella [56], whereas mice in which Shh has been deleted in Purkinje cells (*Shh^c^/Shh^n^;L7-Cre*) have cerebella of reduced complexity [11]. As adults these mice demonstrate decreases in cerebellar volume, short lobules, and a less cellular IGL [11], as do mice with reduced levels of MDM2 (Fig. 1). Mice lacking Gli2, which mediates Shh signaling [45], [57], die before birth, precluding analysis of adult cerebella. However, by E18.5, *Gli2^−/−^* mice exhibit a cerebellum smaller than wild-type with a thinner, less proliferative EGL and diminished foliation, as do newborn, *Mdm2^puro/Δ7-9^* mice. When Gli2 has been conditionally deleted (*Gli2-En1*), mice survive to adulthood with small cerebella, short folia and a diminished IGL[45], [57], as do *Mdm2^puro/Δ7-9^* mice. In contrast to Gli2, mice lacking Gli1 are phenotypically normal [58]. A role for Gli1 in the cerebellum is demonstrated however by the observation that loss of Gli1 further accentuates the foliation defects of the Gli2-deficient cerebellum [45], [57]. Our data show that MDM2 regulates both Gli1 and Gli2 levels in GNPs supporting the concept that attenuated Shh signaling contributes, at least partially, to the foliation defects observed in *Mdm2^puro/Δ7-9^* cerebella.

Impaired foliation of *Mdm2^puro/Δ7-9^* cerebella is p53-dependent thereby implicating p53 in the regulation of Gli1 and Gli2 in GNPs. Recent studies in neural stem cells have demonstrated that p53 inhibits Gli1 in two ways: it reduces the nuclear localization and level of Gli1 protein and promotes phosphorylation of an N-terminally truncated form of Gli1, resulting in a less active isoform [49].

[49]. It is unknown whether p53 similarly inhibits Gli2. The mechanism(s) by which Gli1 and Gli2 is regulated by p53 in GNPs are an important area of future investigation.

The interconnections between the p53 and Shh pathways may be greater than previously surmised. We show that Shh signaling promotes the accumulation and phosphorylation of MDM2 in GNPs. Although the Shh target NMyc binds to the *Mdm2* gene and directly regulate *Mdm2* transcription in neuroblastoma cell lines [59] we find that the level of total *Mdm2* mRNA is not significantly increased following Shh stimulation of GNPs (Fig. 7C,D). The regulation of MDM2 in GNPs also differs from that in C3H10T1/2 and MEFs in which Shh was shown to promote an increase in p-MDM2^S166^ without a concomitant increase in MDM2 protein levels [60]. Thus, Shh signaling appears to regulate MDM2 function through different transcriptional and post-transcriptional mechanisms depending on cell type.

Elevated MDM2 function is predicted to keep basal p53 activity low. Indeed, deregulated SMO in C3H10T1/2 and MEFs promotes MDM2-mediated inhibition of p53 by enhancing the binding of MDM2 to p53, and abrogates p53-mediated growth arrest and apoptosis in response to DNA damage [60]. Conversely, p53-dependent apoptosis is increased in the retina and developing CNS of *Shh^−/−^* zebrafish embryos suggesting that p53 function is actively suppressed by Shh signaling [61]. These findings prompt us to speculate that in cancers in which the deregulation of Shh signaling is the initiating event, p53 function will be automatically inhibited, if only partially, through the enhancement of MDM2 activity by Shh signaling. If so, this mechanism may account for the infrequency of p53 gene mutations in childhood MB tumors. There is growing knowledge that genes important for development are also important for tumorigenesis. The inhibition of p53 through the up-regulation of MDM2 function in development may have broad implications for understanding the etiology of childhood embryonal tumors of the CNS that is thought to have a strong developmental component.

We present a model in which MDM2 is required to down-regulate p53 such that one or more Gli proteins is fully functional to transmit the Shh signal for GNP proliferation and tumorigenesis (Fig. 8E). In support of this model, expression of multiple Shh target genes is decreased in GNPs from mice with reduced levels of MDM2. We find that even a moderate, ∼20% reduction in MDM2 is sufficient to attenuate Shh target gene expression, illustrating that Shh signaling in GNPs is sensitive to the level of MDM2 (Fig. 6A). Despite the attenuation of Shh signaling in mice with either ∼50% or 80% the wild-type level of MDM2, gross defects in cerebellar development were observed only when the level of MDM2 was reduced to ∼30% of the wild-type level. The p53 and Shh pathways influence many physiological processes, including cell proliferation, survival, differentiation, and stem cell renewal [reviewed in 62], [63]. The coordinate regulation of these two signaling pathways may be necessary during critical periods of development, allowing high levels of Shh-mediated proliferation without engaging p53 at a cell cycle checkpoint.

Defective foliation in *Mdm2^puro/Δ7-9^* mice does not result in motor defects as measured here. Our results do not preclude the possibility that *Mdm2* expression may be associated with human cerebellar defects. In people, there is a single nucleotide polymorphism in the *Mdm2* gene that influences both *Mdm2* gene expression and susceptibility to breast and other cancers [64]. It will be of interest to determine whether *Mdm2* gene expression levels are associated with cerebellar hypoplasia or behavioral defects in people. Moreover, information about pathways regulating the ability of MDM2 to inhibit p53 may provide insight into the pathology of some congenital birth defects (e.g. Treacher Collins Syndrome) or chronic human diseases (e.g. Parkinson's disease) for which there is growing evidence that heightened p53 activity in neuronal cells may be an important contributing factor [65], .

While *p53* is the most commonly mutated gene in human cancers, a large subset of tumors with wild-type *p53* overexpress MDM2, an observation that has led to the development of therapeutics designed to block the MDM2-p53 interaction [68]. This study demonstrates MDM2 promotes Shh-induced pre-neoplastic lesions in the cerebellum, suggesting that inhibition of MDM2 may be of high therapeutic value for the prevention or treatment of MB. However, our results also indicate that MDM2-targeted therapeutics may cause developmental defects. If MDM2 is found to be a critical component of pathways regulating MB in people, a cautious approach to MDM2 inhibition may be successful. Our results point to MDM2 as an important mediator of Shh signaling that may contribute to human medulloblastoma and its treatment.

## Supporting Information

Table S1 (DOC)
