# Supplementary material for: The p53 Inhibitor MDM2 Facilitates Sonic Hedgehog-Mediated Tumorigenesis and Influences Cerebellar Foliation
Source: PLoS One. 2011 Mar 18;6(3):e17884. doi: 10.1371/journal.pone.0017884 (PMC3060880; doi:10.1371/journal.pone.0017884)
Supplement: Table S1 — (DOC) [file pone.0017884.s001.doc]

**Supplemental Data:**

To assess whether the structural defects of the cerebellum in *Mdm2^puro/Δ7-9^* mice caused ataxia, loss of coordination or other behavioral defects, a battery of tests were run on 4-month old animals [41]. Initially, the general health of each animal was assessed. *Mdm2^puro/Δ7-9^* mice were smaller, but appeared healthy with the exception of a propensity for bald patches that appeared to be due to barbering (Table 1). All 4-month-old wild-type and mutant mice had normal sensory responses to touches to the ear and whiskers and all mice could perceive “the edge of a cliff”. Neuromuscular strength was normal, as measured by the ability to hang on a wire for 1 minute. Motor control was normal as indicated by the ability of the mice to both right themselves immediately when put on their backs and splay their legs to adjust their posture to the movement of the cage. When observed in an empty cage, mutant mice appeared normal, with the exception that they reared more often than wild-type mice. However, this difference was not statistically significant (*P*=0.18). Mutant mice were not ataxic; the length of stride was similar in wild-type and mutant mice, as was the variation between stride lengths (*P*>0.23 for all parameters). Motor coordination and balance was assessed by measuring the ability of the mice to remain on a drum rotating at a constant speed for 60 seconds and then accelerating over 5 minutes. All the mice stayed on the drum while it was rotating at a constant speed. Unexpectedly, mutants were significantly better at maintaining balance on the accelerating drum than were wild-type mice (*P*<0.001), indicating that motor coordination and balance were not adversely affected by the cerebellar defects in *Mdm2^puro/Δ7-9^* mice. It has been reported that reduced body weight can facilitate Rotarod performance [70]. However, body weight did not correlate with time on the accelerating Rotarod in our cohort (R^2^=0.16). To determine whether motor coordination and balance were impaired in younger mice, 4-week-old mice were assessed in a subset of tests. No differences between 4-month-old and 4-week-old mice were observed. The ability of *Mdm2^puro/Δ7-9^* mice to remain longer on the rotating drum was apparent by the age of 4 weeks (*P*=0.0098). Moreover, by ten days of age, all wild-type and mutant mice could right themselves immediately, although gross histological differences were already evident in the cerebella

Table S1. Behavior analysis of 4-month-old *Mdm2^puro/Δ7-9^* mice

Genotype *Mdm2^+/+^* *Mdm2^puro/Δ7-9^*

Number of mice evaluated 18 17

Physical assessment and simple reflexes (% abnormal)

Bald patches 0 38

Righting reflex 0 0

Whisker response 0 0

Eye blink/ear twitch 0 0

Pupil constriction/dilation 0 0

General observation measurements (%)

Wild running 0 0

Freezing 0 0

Sniffing 67 76

Licking 22 29

Rearing 5.5 18

Cliff behavior (time to edge, seconds)

Male 0.46 0.43

Female 0.64 0.65

Clasping reflex (% abnormal) 0 0

Wire hang test (% abnormal) 0 0

Rotarod test (sec. before dropping)

Male 132 221

Female 144 205

Footprint test (avg. stride/variation)

Male

Left 5.2/1.7 5.5/1.2

Right 5.3/1.6 5.4/1.5 Female

Left 5.2/2.1 4.9/1.5

Right 5.1/2.2 5.0/2.2
